# Anti N-methyl-D-aspartate receptor (NMDAr) encephalitis during pregnancy: A case report

**DOI:** 10.1016/j.ebr.2022.100535

**Published:** 2022-03-19

**Authors:** Fedele Dono, Giacomo Evangelista, Stefano Consoli, Giovanna Scorrano, Mirella Russo, Martina di Pietro, Marco Onofrj, Stefano L. Sensi, Francesca Anzellotti

**Affiliations:** a Department of Neuroscience, Imaging and Clinical Science, “G. d’Annunzio” University of Chieti-Pescara, Italy; b Center of Advance Studies and Technologies (CAST), “G. d’Annunzio” University of Chieti-Pescara, Italy; c Department of Pediatrics, “G. d’Annunzio” University of Chieti-Pescara, Italy; d Epilepsy Center, “SS Annunziata” Hospital, Chieti, Italy

**Keywords:** Maternal outcome, Fetal outcome, Epilepsy, Seizures, Lacosamide

## Abstract

Anti-N-methyl-D-aspartate receptor (NMDAr) antibody encephalitis is an autoimmune disorder characterized by reduced synaptic activity of the NMDAr due to circulating antibodies that target the NR1 subunit. Few cases of anti-NMDAR encephalitis during pregnancy have been described. The permeation of anti-NR1 antibodies through the placenta can be instrumental in the development of complications in newborns. We describe a case of a young woman suffering from anti-NMDAR encephalitis during the first trimester of pregnancy and focus on diagnostic and therapeutic management.

## Highlights

• Focal motor status epilepticus can be a presenting sign of anti-NMDA rencephalitis during pregnancy.
• After the immunomodulatory treatment, the patient showed only attention deficits with normal global cognition.
• The newborn presented normal birth weight kg with APGAR 10 with no sign of fetal distress nor major or minor malformations.

## 1 Introduction

The anti-N-methyl-D-aspartate receptor (NMDAr) antibodies encephalitis is the most frequent autoimmune encephalitis (AE) occurring in young women [1]. This condition is frequently associated with neoplasia, paraneoplastic syndromes, and especially with ovarian teratoma [2]. The autoimmune etiology is characterized by synaptic NMDAr dysfunction driven by antibodies targeting the receptor NR1 subunit [3].

Few cases of anti-NMDAR encephalitis during pregnancy have been described [5]. The permeation of anti-NR1 antibodies through the placenta as well as the mother symptoms may be crucial for the development of complications in newborns [6]. In treating this condition, the clinician must consider the teratogenic and toxic effects of treatments on the fetus and balance them with benefits for the mother. Especially in the first trimester of pregnancy, the use of anti-seizure medication (i.e. carbamazepine and phenytoin), immunomodulatory drugs (i.e., cyclophosphamide), or the radiological assessment of any underlying neoplasia (i.e., computerized tomography of the abdomen and pelvis with contrast enhancement for ovarian teratoma) are associated with increased rates of congenital malformations (like spina bifida and cardiac anomalies) or newborn distress [7].

In the present report, we describe the case of a young woman suffering from anti-NMDAR encephalitis during the first trimester of pregnancy. We aim to highlight the positive maternal and fetal outcome and focus on diagnostic and therapeutic management.

## 2 Case presentation

A 29-year-old woman in the 7th gestational week came to our observation for the sudden onset of continuous, ongoing, focal motor seizures involving the right side of the face. According to her past medical history, in the previous seven days, the patient had presented several episodes of emotional liability with sudden changes in her mood and behavior (i.e., uncontrolled lapses of crying or laughing). The medical history was negative for any significant comorbidity.

**Fig. 1:**
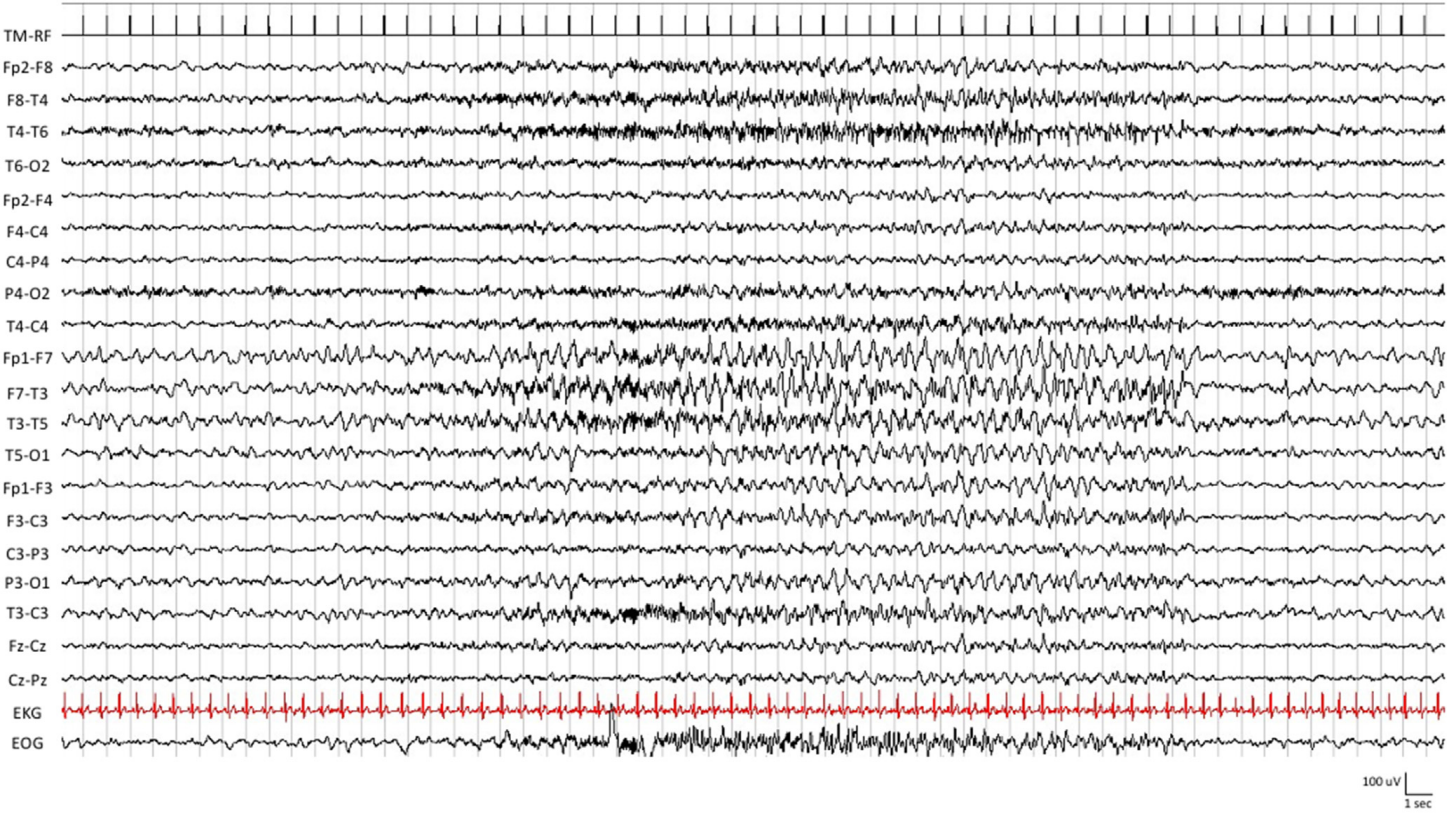
Electroencephalogram (EEG) findings. The patient EEG, recorded in the acute phase two days after the admission, shows continuous high-amplitude rhythmic 3–5 Hz slow waves and sporadic diphasic sharp waves over the left fronto-centro-temporal derivations.

At admission to the Emergency Room, during the neurological evaluation, the patient showed continuous (lasting > 60 min), stereotyped, rhythmic muscle jerks involving the right labial commissure and sialorrhea. The patient was fully aware and did not show other focal neurological signs. The patient underwent a video-electroencephalogram (video-EEG) recording, which showed continuous high-amplitude rhythmic 3–5 Hz slow waves and sporadic biphasic sharp waves over the left fronto-centro-temporal derivations (Fig. 1). A diagnosis of focal motor status epilepticus was made according to the International League Against Epilepsy diagnostic criteria [8]. The patient was treated with two boluses of intravenous (IV) lorazepam (4 mg) followed by an IV bolus of levetiracetam (1000 mg) that produced a good electroclinical response. An anti-seizure medication (ASM) course with levetiracetam (1000 mg twice a day) was then started. Soon after, magnetic resonance imaging (MRI) of the brain without contrast and a magnetic resonance angiography (MRA) of the intracranial vessels were performed and revealed no abnormalities. The patient also underwent an obstetric evaluation with ultrasound fetal echography that was negative.

**Fig. 2:**
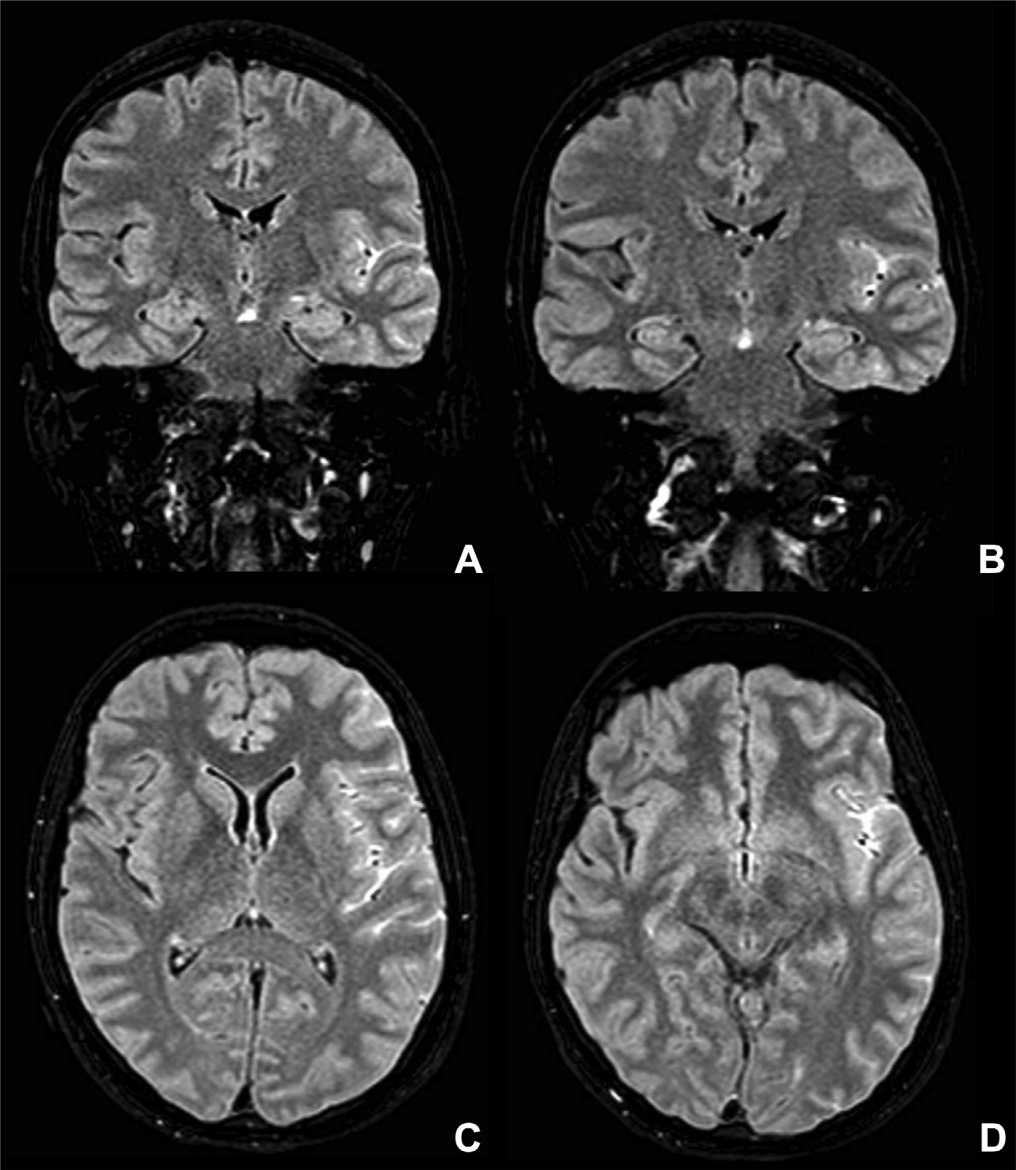
Magnetic Resonance Imaging (MRI) brain findings The panel depicts the patient’s MRI scans taken (performed in the acute phase two days after the admission). A) and B) coronal Fluid-attenuated Inversion Recovery (FLAIR) T2-weighted images, C) and D) axial FLAIR T2-weighted images show hyperintense alterations in the left temporo-fronto-parietal cortex.

In the following 72 hours, a worsening of the clinical picture was observed. In particular, there was an increased frequency of the aforementioned focal motor seizures, with some episodes eventually evolving in focal-to-bilateral tonic-clonic seizures. The patient also presented psychomotor agitation with non-finalistic movements, aggression, and mutism. The patient was monitored with continuous EEG, which showed continuous high-amplitude rhythmic 3–5 Hz slow waves and sporadic diphasic sharp waves over the left fronto-centro-temporal derivations. Additional brain MRI scans were performed, which now showed hyperintense alterations over the left temporo-fronto-parietal cortex in fluid-attenuated inversion recovery T2-weighted sequences (Fig. 2). No pathological findings were detected in the brain MRA of intracranial vessels. A CT scan of the brain was also performed to exclude subarachnoid hemorrhage, which resulted negative. The CT scan was performed using a lead cover over the abdomen to reduce fetal exposure to radiation. A lumbar puncture was then performed, which showed mild lymphocytic pleocytosis (white blood cells: 8 cells/mm^3^) with increased protein (70.5 mg/dl) and normal glucose levels. A polymerase chain reaction assessment of neurotropic viruses and the search of oligoclonal bands were negative. However, the antibodies panel for AE revealed high levels of anti-NMDAR antibodies in the cerebrospinal fluid, a finding confirmed in the serum (Table 1). A diagnosis of anti-NMDAr AE was made in line with the Grauss criteria [9]. ASM therapy was implemented with lacosamide (100 mg twice a day) to avoid relapses of bilateral tonic-clonic seizures. In contrast, for the underlying encephalitis, an immunomodulant course with high-dose steroid (prednisone 1 g per 5 days followed by oral prednisolone 60 mg/day for 10 days) and subsequent plasmapheresis (PLEX) therapy (5 cycles in 10 days) were performed. This combined treatment produced a progressive improvement of the clinical picture. The patient also underwent pelvic and abdomen MRI, ovarian echography, and chest-RX, all results were negative. Blood tests (i.e., white and red blood cell counts, white blood cells formula and serum electrolytes) were also negative.

**Table 1:** Cerebrospinal fluid (CSF) and serum analysis.

*Cytochemical examination*	*Result*	*Autoimmune panel*	*Titer*
**Cerebrospinal fluid analysis**			
*Appearance*	Clear	*Anti-Ca^2+^ Channel Ab*	Negative
*White cells*	8 cells/mm^3^	*Aanti-VGCK Ab*	Negative
*Glucose*	66 mg/dl	*Anti-GLUR3 Ab*	Negative
*Proteins*	70.5 mg/dl	*Anti-AMPA1,2 Ab*	Negative
***Microbiological panel***		*Anti-CASPR2 Ab*	Negative
*HSV-1*	Negative	*Anti-LGI1 Ab*	Negative
*HSV-2*	Negative	*Anti-NMDAR Ab*	***1:32***
*HHV-6*	Negative	*Anti-GABA Ab*	Negative
*HHV-7*	Negative	*Anti-GAD65 Ab*	Negative
*HHV-8*	Negative	*Anti-MOG Ab*	Negative
*CMV*	Negative	*Anti-AQP4 Ab*	Negative
*EBV*	Negative	*Oligoclonal bands*	Negative
*VZV*	Negative		

**Serum analysis**			
***Autoimmune panel***	***Titer***		
*Anti-Ca^2+^ Channel Ab*	Negative	Anti-Ma1 Ab	Negative
*Anti-VGCK Ab*	Negative	Anti-Ma2/Ta Ab	Negative
*Anti-GLUR3 Ab*	Negative	Anti-CV2 Ab	Negative
*Anti-AMPA1,2 Ab*	Negative	Anti-Hu Ab	Negative
*Anti-CASPR2 Ab*	Negative	Anti-Ri p54 Ab	Negative
*Anti-LGI1 Ab*	Negative	Anti-Yo Ab	Negative
*Anti-NMDAR Ab*	***1:32***	Anti-recoverin Ab	Negative
*Anti-GABA Ab*	Negative	Anti-amphiphysin Ab	Negative
*Anti-GAD65 Ab*	Negative	Anti-SOX1 Ab	Negative
*ANA*	Negative	Anti-Zic4 Ab	Negative
*ENA*	Negative	Anti-titin Ab	Negative
*Antiphospholipid antibodies*	Negative	Anti-Tr Ab	Negative

At the 12th gestational week, the fetal ultrasound showed standard biparietal and cerebellar indices, normal abdomen dimension, and heart kinetic. The patient underwent neuropsychological evaluations revealing the presence of attention and language deficits (decreased fluency, palilalia and echolalia), with normal global cognition.

During the following weeks of pregnancy, the patient did not experience any relapse of the neurological and psychiatric manifestations. At the 39th week of gestational age, natural delivery was performed. At the delivery, the newborn presented normal birth weight (2.800 kg) with APGAR 9–10 at the first and the fifth minute. The newborn did not present any sign of fetal distress with no major or minor malformations. The patient decided to avoid breastfeeding. At the follow-up visit, performed 3 months later, the patients did not show abnormalities at the neurological examination. An MRI scan with contrast of the brain indicated complete resolution of the neuroradiological anomalies. The EEG was also unremarkable. At the new neuropsychological evaluations, only attention deficits with normal global cognition were observed. The patients started a slow down-titration of the ASM therapy with the aim to stop the treatment in the following 3 months. In addition, a follow-up total-body CT scan with contrast, pelvic ultrasonography and breast RX were performed, all negative.

## 3 Discussion

Anti-NMDAR encephalitis is one of the most frequent autoimmune epilepsies (AE) during pregnancy [10]. According to the literature, the anti-NR1 antibodies can transfer from the mother to the fetus starting at the 13th week of gestation and increasing during the third trimester. Experimental models show that maternal-fetal autoantibodies transfer can cause harmful consequences on the fetus. In previous reports, placental antibodies transfer has been confirmed in a few cases (Table 2). The presence of anti-NR1 antibodies in the newborn was not always associated with detectable neurological deficits, which perhaps are the result of a combination of factors that may include the potential pathogenic effects of the antibodies (usually transient) along with the side effects of sedatives, ASM, and other drugs used for the treatment of AE. In our report we did not test anti-NR1 antibodies serum titer in the newborn due to the absence of any clinical manifestations after the delivery as well as in the following weeks.

**Table 2:** Literature revision of the cases of anti-NMDAR autoimmune encephalitis during pregnancy with maternal and fetal outcome. IV: intravenous; IVIg: intravenous immunoglobulin; RTX: rituximab; PLEX: plasmapheresis; C-section: caesarean section.

Reference	Age	Gestational age (weeks)	Presenting symptoms	Teratoma	Neonatal antibodies	Treatment	Outcome: mother	Outcome: baby
Ito et al. [22] 2010	19	17	Dyskinesia, behavior abnormalities	No	Not tested	Corticosteroids	Normal	Emergency C-section at 33 weeks gestation. Normal baby
Kumar et al. [23] 2010	19	17	Behavior abnormalities	No	Not tested	IV Methylprednisolone	Normal	Normal vaginal delivery at 37 weeks gestation. Normal baby
Kumar et al. [23] 2010	20	8	Behavior abnormalities	No	Not tested	IVIg	Minimal deficits	Termination of pregnancy at 10 weeks
Kumar et al. [23] 2010	19	14	Headache followed by behavior abnormalities	Yes	Not tested	IVIg IV Methylprednisolone Resection of teratoma	Both mother and baby survived-C-section delivery at 38 weeks gestation	Normal
McCarthy et al. [24] 2012	32	8	Autonomic symptoms, behavior abnormalities	Yes	No	IV-Methylprednisolone PLEX Resection of teratoma	Normal	C-section delivery at 32 weeks. Normal baby
Jagota et al. [25] 2014	18	9	Orolingual movements, eye deviation, fever	No	Yes	Azathioprine IVIg	Patient died due to infection. Baby survived, delivered at 34 weeks (NVD) Baby-Delayed in global development, seizures	Global developmental delay. Seizure. Cortical dysplasia
Lamale-Smith et al. [26] 2015	24	20	Catatonia, disoriented, confused	No	Yes	IV Methylprednisolone IVIg	Disinhibition, memory impairment	C-section at 28 weeks. Normal
Chan et al. [27] 2015	23	1st semester	Fever, hallucinations, disinhibited behavior, confusion	Yes	Not tested	IV-Methylprednisolone PLEX Rituximab Resection of teratoma	Normal	Miscarriage
Mathis Stephanie et al. [28] 2015	21	10	Behavior abnormalities	No	Not tested	IV-Methylpredinisolone IVIg	Slight memory impairment	Normal vaginal delivery at 40 weeks gestation
Kim et al. [29] 2015	28	7	Abnormal behavior, hypoventilation, dyskinesia and epileptic seizure	Yes	Not tested	IV-Methylprednisolone IVIg Oral corticosteroids PLEX, RTX, Resection of teratoma	Slight cognitive function deficits	Miscarriage
Xiao et al. [30] 2017	24	28	Psychiatric symptoms –visual and auditory hallucinations	No	Not tested	IVIg IV-Methylprednisolone Bilateral ovarian wedge resection	Normal	Emergency C-section at 33 weeks gestation. Normal baby
Shanani et al. [31] 2015	26	22	Headache, behavioral abnormalities	No	Not tested	Oral corticosteroids IV-Methylprednisolone, PLEX	Normal	Normal vaginal delivery. Normal baby
Joubert et al. [20] 2020	19	25	Visual hallucination, delirium and agitation	Yes	Not tested	IVIg, cyclophosphamide, Resection of teratoma	Poor responder	C-section. Normal baby
Joubert et al. [20] 2020	37	33	Bulbar palsy and hemifacial sensitivity deficit	No	Not tested	IVIg, cyclophosphamide	Normal	C-section. Normal baby
Joubert et Al. [20] 2020	31	20	Orofacial dyskinesia, cognitive fluctuation, memory deficits, delirium	Yes	Not tested	IVIg, RTX, cyclophosphamide, Resection of teratoma	Poor responder	C-section. Normal baby
Joubert et al. [20] 2020	25	5	Epilepsy and behavior abnormalities	No	Not tested	IVIg, PLEX, RTX	Poor responder	Prematurity
Joubert et al. [20] 2020	20	12	Behavioral abnormalities, dysarthria, motor aphasia	No	Not tested	IVIg	Normal	Normal vaginal delivery. Normal baby
Joubert et al. [20] 2020	23	8	Nausea, visual hallucination, delirium	Yes	Not tested	IVIg, Resection of teratoma	Normal	C-section. Low birth weigh
Keskin et al. [32] 2019	27	18	Seizure, headache, visual hallucination	No	Not tested	IVIg IV-Methylprednisolone, PLEX	Death	Death
Jung et al. [33] 2020	28	24	Depression, focal seizure, headache	No	Not tested	IVIg Oral corticosteroids IV-Methylprednisolone RTX	Normal	C-section. Normal baby
Tailland et al. [34] 2020	37	18	Orofacial dyskinesia, pyramidal bilateral syndrome	No	Not tested	IVIg IV-Methylprednisolone	Not available	Normal

The diagnostic and therapeutic management of anti-NMDAr encephalitis during pregnancy is particularly challenging due to the possible fetal side effects of diagnostic and therapeutic interventions. The diagnosis of AE is clinical [4], but instrumental investigations like radiological imaging is supportive and important for a correct differential diagnosis. Some concerns have been raised about using radiation-based techniques (i.e., x-ray procedures and CT scans with or without iodinated contrast) for their potential teratogenic effects whereas the use of MRI scans or ultrasonography has not been associated with an increased risk of fetal malformation. However fetal safety has been questioned if MRI scan is performed during the first trimester of pregnancy. According to the latest evidence, exposure to MRI during the first trimester of pregnancy is not associated with an increased risk of harm to the fetus or in early childhood. On the other hand, gadolinium MRI at any time during pregnancy can have teratogenic effects and is associated with an increased risk of a broad set of rheumatological or inflammatory newborn complications as well as stillbirth or neonatal death [11]. In considering available data and the risk of teratogenicity, the American College of Radiology concludes that no special consideration is recommended for the first (versus any other) trimester in pregnancy [12]. In our case, the diagnosis of AE and the search of underlying neoplasia were performed by MRI scans and radiation techniques without contrast and employing a low-exposure radiation approach. In addition, the use of a lead cover over the abdomen helped prevent fetal exposure to radiation during CT scans, even though the quantity of radiation was further below the dangerous exposure level. A total-body CT scan with iodinated contrast and pelvic ultrasonography was performed after delivery to exclude the presence of neoplasms.

Around 55% of women suffering from AE have an ovarian teratoma, but other tumors and hematological diseases have also been described. As reported in Table 2, several cases of AE during pregnancy associated with ovarian teratoma have been described. However, our report stresses that AE onset during pregnancy is not necessarily associated with underlying tumor pathology, though screening for neoplasms is essential. The range and frequency of associated malignancies differ according to the autoantibody detected. The early detection of tumors is important for the prognosis but is also critical as immunotherapy could hamper tumor detection. If the first neoplastic screening is negative, further assessment should be repeated every 4–6 months for 2 years [13]. However, the recommendation should be personalized according to risk factors, clinical evolution, and medical resources.

Concerning therapy, it is well known that some ASMs and immunomodulatory treatments can have teratogenic and toxic effects. Evidence indicates that certain ASMs, especially first-generation drugs (phenytoin or phenobarbital) are associated with increased rates of congenital malformations (spina bifida, cardiac anomalies) as well as with newborn distress [7]. In contrast, new-generation ASMs seem to exhibit a safer profile [14]. The risk of major congenital malformations has been confirmed for valproic acid [14], whereas no consistent association with major congenital malformation has been indicated for lamotrigine, levetiracetam, carbamazepine, oxcarbazepine, gabapentin, and lacosamide [15]. Fetal valproate exposure poses a dose-dependent risk for malformations that exceeds other antiseizure medications. Teratogens act in a dose-dependent manner and some studies suggest that even some of our apparently safer ASM in regard to malformations appear to have dose-dependent risks [14], [16].

Even though seizures in AE are characteristically resistant to ASMs, these drugs maintain a role for symptomatic management. A recent study [17] tried to evaluate seizure responses to immunotherapy and ASM in patients with AE. Interestingly, ASM treatment (even after changing dosage or treatment regimen) was associated with low seizure control and, in some patients, the use of ASM was followed by serious psychiatric manifestations (i.e. behavioral changes, psychosis and suicidal thoughts). On the other hand, patients treated with immunotherapy resulted in seizure freedom faster (28 days from the start of the treatment) than ASM (59 days from the start of the treatment) and also more often (53% of patients) than ASM (14% of patients). Patients treated earlier in the disease course with immunotherapy were seizure-free quicker. Thus, ASM should be considered as an add-on treatment to immunotherapy in order to better achieve seizure freedom. Furthermore, a recent retrospective study demonstrated a considerably higher efficacy of sodium channel blocking compounds to produce seizure freedom in AE patients. In our case, we administered ASM (in particular the sodium channel blocker lacosamide) to prevent the onset of focal-to-bilateral tonic-clonic seizures, a condition that could have been detrimental to fetal well-being. After the delivery, in line with the aforementioned evidence, we aimed to stop ASM in the following 3 months.

Immunomodulatory treatment with systemic corticosteroids seems to be safe during pregnancy. Indeed, pregnant women receiving corticosteroids usually show a low risk of developing major congenital malformations, even though premature rupture of amniotic membranes and low birth weight babies may occur [18]. Some authors indicated an increased risk of preterm birth, small for gestational age, low birth weight, intrauterine growth restriction and neonatal intensive care unit admission [18]. Reports describing the use of PLEX during pregnancy are limited. However, consensus reports suggest that PLEX is safe and appropriate, especially in patients who have failed other immunomodulatory treatments. PLEX can be used safely during pregnancy with the proper training of a multidisciplinary team. Intravenous immunoglobulins (IVIg) are also safe during pregnancy. The obstetric literature contains numerous reports of intravenous immunoglobulin therapy for various conditions encountered during pregnancy [19]. Furthermore, several case reports and case series (Table 2) showed a favorable outcome in pregnancy patients with AE treated with IVIg with a low occurrence of side effects as well as infectious morbidity. On the other hand, the use of the chimeric anti-CD20 monoclonal antibody rituximab as third-line immunomodulatory treatment in non-responder AE during pregnancy is controversial. Rituximab is a pregnancy category C medication and should be stopped 12 months prior to attempting conception. Even though anecdotical cases are reported in the literature [20], safety and efficacy data about rituximab in the treatment of AE during pregnancy are not available. A recent study [21] based on a global database encountering 231 pregnant women treated with rituximab showed that of 153 pregnancies with known outcomes, 90 resulted in live births. However, the study reported 22 infants born prematurely, 11 neonates with hematologic abnormalities and 4 neonatal with systemic infections. In addition, 2 congenital malformations (i.e.: clubfoot in one twin, and cardiac malformation in a singleton birth) were identified.

In our case, the treatment with steroids and PLEX produced complete relief of the symptoms, good clinical response, and no evidence of newborn’s malformation. In line with previous reports (Table 2), treatment with steroids and PLEX has been associated with a good clinical outcome for the newborn and mother. In addition, the follow-up evaluation showed sustained benefits with full remission of the neuropsychological deficits.

## 4 Conclusion

Our case describes the diagnostic and therapeutic management of a patient suffering from anti-NMDAr encephalitis during pregnancy. Immunomodulatory treatments along with ASM have played a key role in control of focal-to-bilateral seizures. The prompt diagnosis and the adequate treatment led to a complete recovery of the mother and a good newborn outcome.

## 5 Statement of ethics

Written informed consent was obtained from the patient for publication of this case report and any accompanying images. The paper is exempt from ethical committee approval because it is not necessary to publish the case report.

## Funding sources

Not applicable.

## Author contributions

FD contributed to the conception and design of the study. FD and SLS wrote the manuscript. GE, SC, MR, MDP, MO, and FA contributed to manuscript revisions, read and approved the submitted version.

## Data availability statement

The data are available from the corresponding author upon reasonable request.

## Conflict of interest statement

The authors declare no conflict of interest.
